# Comparative efficacy and safety of febuxostat and allopurinol in chronic kidney disease stage 3–5 patients with asymptomatic hyperuricemia: a network meta-analysis

**DOI:** 10.1080/0886022X.2025.2470478

**Published:** 2025-02-27

**Authors:** Jiaojiao Chen, Yanyun Zhang, Yinglin Wang, Lu Chen

**Affiliations:** a Department of Pharmacy, Yantai Yuhuangding Hospital, Shandong, China; b Department of Physical Examination Center, Yantai Yuhuangding Hospital, Shandong, China

**Keywords:** Chronic kidney disease, asymptomatic hyperuricemia, febuxostat, allopurinol, renal function, safety profile

## Abstract

**Objective:**

This study evaluates and compares the effectiveness and safety of febuxostat and allopurinol in chronic kidney disease (CKD) stages 3–5 patients with asymptomatic hyperuricemia using a network meta-analysis.

**Methods:**

A systematic review and network meta-analysis were conducted, adhering to PRISMA-NMA guidelines. Searches included PubMed, Embase, Cochrane Library, and Chinese databases up to June 2024. Randomized controlled trials (RCTs) and cohort studies were assessed for methodological rigor using GRADE.

**Results:**

A total of 12 RCTs and 4 cohort studies (*n* = 2,423 participants) were included. Febuxostat was associated with greater improvements in estimated glomerular filtration rate compared to allopurinol (MD, 4.99 mL/min/1.73 m^2^; 95%CI −0.65 to 10.78; certainty: low) and placebo (MD, 4.72 mL/min/1.73 m^2^; 95%CI 0.67 to 8.82; low). Serum uric acid reduction was also more pronounced with febuxostat (MD, −0.61 mg/dL; 95%CI −1.15 to −0.05; moderate). Safety outcomes, including major cardiovascular events and adverse events, showed no significant differences between febuxostat and allopurinol. Subgroup analyses revealed enhanced effectiveness of febuxostat at six months of treatment.

**Conclusions:**

This analysis provides robust evidence that febuxostat might offers greater improvements in kidney function and uric acid levels compared to allopurinol or placebo in asymptomatic hyperuricemia with CKD stage 3–5 patients, without compromising safety. These findings can guide clinical decision-making and treatment optimization.

## 1. Introduction

Hyperuricemia is widespread among patients with chronic kidney disease (CKD) and increases in severity as kidney function declines [1,2]. It has been proven as an independent risk factor for newly diagnosed CKD and for the deterioration of kidney function in existing CKD patients, potentially predicting premature death [3–6]. Urate-lowering therapy (ULT) for symptomatic hyperuricemia, such as gout, tophi, and kidney stones has been shown to have potential benefits on cardiovascular and renal outcomes [7–9]. Xanthine oxidase inhibitors are recommended by guidelines as the first-line ULT interventions for treating chronic gout with hyperuricemia [10–12].

Currently, an increasing number of hyperuricemic patients remain asymptomatic, defined as the absence of either gout flares, tophaceous gout, acute or chronic hyperuricemic nephropathy, or uric acid nephrolithiasis [13]. Due to the side effects of urate-lowering drugs, the necessity of ULT for patients with asymptomatic hyperuricemia, particularly those with CKD stages 3–5, remains controversial [10–12,14–17]. The American College of Rheumatology Guideline for the Management of Gout [10] does not recommend initiating ULT for patients with asymptomatic hyperuricemia, as the benefits of ULT may not outweigh the potential treatment costs. In contrast, the guidelines of the Japanese and Chinese Society of Gout [15,18] both recommended pharmacological treatment for asymptomatic hyperuricemia patients with CKD to retard the decline in kidney function. The results from the recent meta-analysis have demonstrated that allopurinol or febuxostat may be the optimal ULTs in reducing composite renal events and improving renal function without significantly increasing the risk of adverse events (AEs) in asymptomatic hyperuricemia patients [19,20]. Nevertheless, most previous published trials compared these drugs to placebo/no treatments, with a lack of head-to-head studies, leaving clinicians and patients uncertain about the magnitude of their benefits [21–24].

This study aims to conduct a network meta-analysis (NMA) to comprehensively compare the relative efficacy and safety of allopurinol and febuxostat in CKD stage 3–5 patients with asymptomatic hyperuricemia. The findings are expected to assist in clinical decision-making and support the development of optimal treatment strategies for asymptomatic hyperuricemia in patients with CKD stage 3–5 in the future.

## 2. Methods

The NMA was performed in accordance with Preferred Reporting Items for Systematic Review and Meta-Analysis extension statement for NMA (Supplementary Table S1) [25]. This study was registered in PROSPERO (CRD42024499551).

### 2.1. Search strategy and selection criteria

PubMed, Embase, the Cochrane Central Register of Controlled Trials, ClinicalTrials.gov, China National Knowledge Infrastructure, WanFang database, and SinoMed database were searched from inception to June 4th 2024, using the term ‘hyperuricemia’, ‘chronic renal insufficiencies’, ‘febuxostat’, ‘allopurinol’, and their synonyms shown in Supplementary Table S2. Additionally, a manual search of reference lists of relevant studies was performed to identify further eligible studies. We identified randomized controlled trials (RCTs) and cohort studies comparing febuxostat or allopurinol with placebo or another urate lowering therapy in CKD stages 3–5 patients complicated with asymptomatic hyperuricemia, and no languages restrictions.

### 2.2. Eligibility criteria

Inclusion criteria: adults (≥18 years old) CKD 3–5 patients (estimated glomerular filtration rate/eGFR <60 mL/min/1.73 m^2^) with hyperuricemia (serum uric acid/SUA concentration ≥6.8 mg/dl [420 µmol/L] in men or ≥6.0 mg/dl [360 µmol/L] in women), or at least mean baseline SUA ≥ 6.0 mg/dl and no symptoms or signs of gouty arthritis, tophi, subcutaneous gouty stones, uric acid kidney stone, or gouty nephropathy.

Exclusion criteria: we excluded patients who met any one of the following criteria: (1) renal transplantation recipients or severe heart disease; (2) pregnancy or lactation; (3) allergies or intolerance to research drugs; (4) secondary hyperuricemia caused by diseases such as tumors or hematological disorders; (5) patients with prior gout flares or acute gout flares; and (6) patients receiving immunosuppressants within the past 3 months.

The primary outcomes were the changes in eGFR from baseline to the end of follow-up and the risk of major adverse cardiovascular events (MACE). The secondary outcomes included the changes in the SUA and urinary albumin/creatinine ratio (ACR) from baseline to the end of follow-up, as well as the risk of drug-related adverse events (AEs).

### 2.3. Data extraction

Two researchers (C.J.J. and Z.Y.Y.) independently screened each trial by reviewing titles, abstracts, and full text using standardized and piloted forms. The baseline information was extracted, including the publication information (publication year, first author, countries, and study type) and trial information (sample size, age, gender, intervention/comparator, duration of study, CKD stage, and the baseline of eGFR and SUA). Discrepancies were resolved through discussion or by the third researcher (C.L. or W.Y.L).

### 2.4. Quality assessment

Two researchers (C.J.J. and Z.Y.Y.) independently assessed the risk of bias of RCTs and cohort studies by using the Cochrane Collaboration’s Risk of Bias 2 (RoB V.2.0) tool [26] and Newcastle-Ottawa Scale (NOS) [27], respectively. Each study was classified as low risk, some concerns, or high risk. The same reviewers assessed the quality of evidence by using the Grading of Recommendations Assessment, Development and Evaluation framework. The quality of evidence was classified into four levels (high, moderate, low, and very low) [28]. All analysis and summaries regarding confidence rating assessments about RCTs were made by University of Berna free CINeMA tools [29,30]. Any discrepancies were resolved through discussion or by the third researcher (C.L. or W.Y.L.).

### 2.5. Statistical analysis

We performed NMA using Bayesian random effect models for interventions that connected to an evidence network by data available from ≥ 2 studies. For dichotomous outcomes, we estimated the results using odds ratio (OR) with 95% confidence intervals (CI). For continuous outcomes, the mean differences (MD) with 95% CI were used. If in the original trials results were available only as graphs, the WebPlotDigitizer (version 4.8, https://apps.automeris.io/wpd4/) was used to transform them to numeric values. When necessary, standard deviations (SD) were calculated according to the Cochrane Handbook for Systemic Review. The Q test and *I*^2^ statistic were utilized to assess heterogeneity among studies. Heterogeneity was deemed significant when *p* < 0.10 and *I*^2^ was 50% or greater [31]. Both direct and indirect comparisons between any pair of comparators (existing closed loops) employed the node-splitting approach to examine consistency. For outcomes without adequate network structure, we performed pairwise meta-analyses only or descriptive analyses were conducted if there was insufficient similarity information to pool the data. Within the Bayesian framework, all interventions were ranked using the surface under the cumulative ranking (SUCRA) curve [32]. Descriptive analyses were conducted if there were insufficient similarity information to pool data.

Subgroup analyses were performed by CKD stage, the dosage of drug, and duration of study when sufficient information was available. A meta-regression method was employed to analyze differences in baseline characteristics. Additionally, sensitivity analysis was performed by excluding high risk of bias of studies. A comparison-adjusted funnel plot and Egger test were used to evaluate small-study effects for individual outcomes when at least 10 eligible studies were available [33]. Statistical significance was set at *p* < 0.05. All NMAs were performed using OpenBUGS version 3.2.3 and the Stata software version 15.0.

## 3. Results

### 3.1. Study selection and characteristics

**Figure 1.**
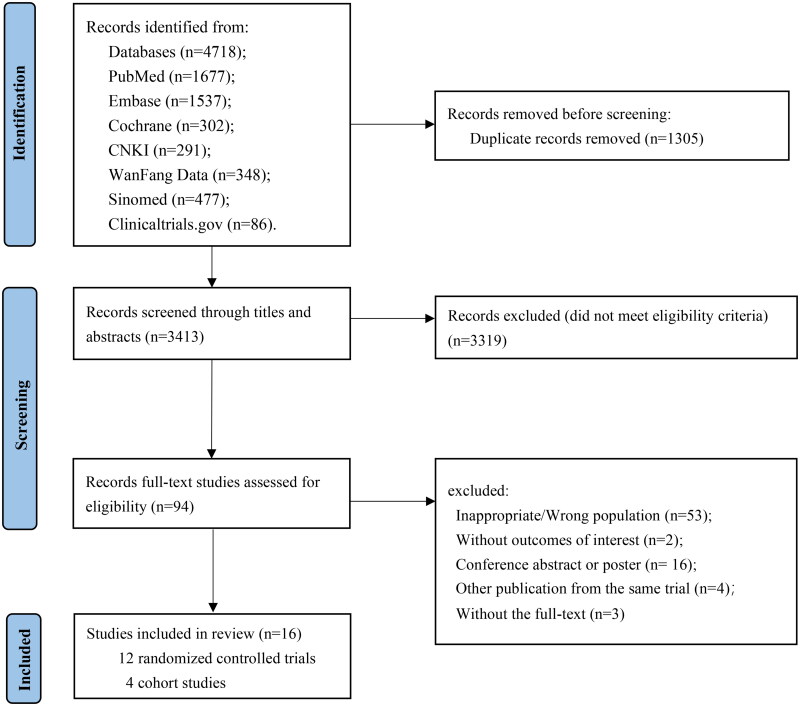
Study selection process.

**Figure 2.**
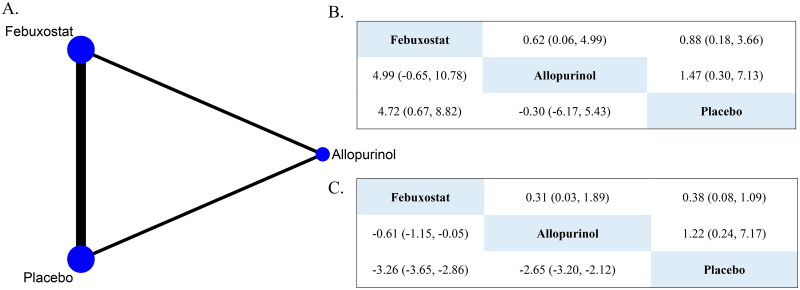
(A) Network diagrams of comparisons on outcomes. (B) Results of Bayesian network meta-analysis of the primary outcomes by RCTs (the upper triangle showed the risk of MACE, and the lower triangle showed the change in eGFR). (C) Results of Bayesian network meta-analysis of the secondary outcomes by RCTs (the upper triangle showed the risk of overall AEs, and the lower triangle showed the change in SUA).

The search strategy identified 4,718 records, of which 16 studies (12 RCTs, 4 cohort studies) met the inclusion criteria, with a total of 2,423 participants enrolled to receive febuxostat, allopurinol, and placebo. The study selection process is illustrated in Figure 1. The baseline characteristics of the included studies are summarized in Table 1. Among these, all studies were double-arm trials, 11 (68.7%) studies were NS-controlled, 5 (31.3%) studies compared febuxostat with allopurinol. The mean sample size of the included studies was 151 patients (range: 38 to 441), and the mean age was 61.5 years (standard deviation: 9.5). The mean duration of study was 8 months, ranged from 2 to 27 months. The dosages of the drugs in most studies were increased gradually and not uniformly, for example, the highest dose of febuxostat was mostly 40 mg/d, and allopurinol was mostly 100 mg/d. Figure 2(A) shows the network of eligible comparisons for outcome.

**Table 1.** Basic characteristics of the included studies.

First author (year)	Study type	Country	Treatment/control	Sample size (T/C)	Demographics				Clinical parameters		
					Age (mean ± SD)	Gender (T/C, Female%)	Duration of study	CKD stage	Baseline serum uric acid (mg/dl)	Baseline eGFR/m L·min-1·(1.73 m2) −1	eGFR formulation
Sircar 2015 [34]	RCT	India	Febuxostat Placebo	45/48	56.22 ± 10.87 58.42 ± 14.52	36%/23%	6 months	3–4	9.0 ± 2.0 8.2 ± 1.1	31.5 ± 13.6 32.6 ± 11.4	MDRD
Jalal 2017 [35]	RCT	America	Allopurinol Placebo	39/41	55.9 ± 13.7 58.9 ± 9.3	18%/22%	12 weeks	3	8.3 ± 1.4 8.7 ± 1.6	41.3 ± 8.9 42.4 ± 9.6	CKD-EPI
Golmohammadi 2017 [36]	RCT	Iran	Allopurinol Placebo	96/100	>18	44.8%/46%	12 months	3–4	>6	15–60	NR
Mukri 2018 [37]	RCT	Malaysia	Febuxostat Placebo	47/46	64 (10) * 67 (6) *	47%/46%	6 months	3–4	9.12 ± 1.70 9.09 ± 1.21	26.2 (14.3) * 28.2 (19.8) *	CKD-EPI
Kimura 2018 [21]	RCT	Japan	Febuxostat Placebo	219/222	65.3 ± 11.8 65.4 ± 12.3	22.4%/23%	108 weeks	3	7.8 ± 0.9 7.8 ± 0.9	45.2 ± 9.5 44.9 ± 9.7	MDRD
Badve 2020 [22]	RCT	Australia and New Zealand	Allopurinol Placebo	182/181	62.3 ± 12.6 62.6 ± 12.9	38%/36%	104 weeks	3–4	8.2 ± 1.8 8.2 ± 1.7	31.6 ± 11.7 31.9 ± 12.4	CKD-EPI
Perrenoud 2020 [38]	RCT	America	Allopurinol Placebo	33/36	59 ± 12 58 ± 9	18%/20%	12 weeks	3	NR	41.4 ± 9.3 41.7 ± 9.3	CKD-EPI
Wen 2020 [39]	RCT	China	Febuxostat Placebo	18/20	58.73 ± 11.50 57.46 ± 10.96	11.11%/15%	24 weeks	3	7.52 ± 1.40 7.12 ± 0.86	45.3 ± 10.6 46.8 ± 9.0	CKD-EPI
Yang 2022 [23]	RCT	China	Allopurinol Febuxostat	37/39	62.2 ± 9.2 61.7 ± 6.9	25%/28.3%	6 months	3	9.59 ± 1.56 9.71 ± 1.76	45.14 ± 10.0 44.29 ± 8.8	CKD-EPI
Yang 2023 [24]	RCT	China	Febuxostat Placebo	47/45	57.0 ± 13.6 56.1 ± 13.2	19.1%/35.6%	12 months	3–4	8.71 ± 1.72 7.92 ± 0.89	29.9 ± 10.8 32.6 ± 8.7	CKD-EPI
Matta 2023 [40]	RCT	India	Allopurinol Febuxostat	55/55	44.87 ± 13.08 45.25 ± 11.51	60%/32.7%	4 months	3–5	10.47 ± 2.21 10.23 ± 2.79	22.85 ± 4.30 23.10 ± 5.23	NR
Nata 2023 [41]	RCT	Thailand	Febuxostat Placebo	42/42	68.0 ± 15.6 65.4 ± 15.1	30%/36.4%	8 weeks	3–4	8.90 ± 1.35 8.39 ± 1.37	31.2 ± 10.2 31.8 ± 14.3	CKD-EPI
Zhang 2019 [42]	Prospective cohort study	China	Allopurinol Febuxostat	55/43	61.1 ± 9.0 62.6 ± 7.0	23.5%/24.5%	6 months	3	9.57 ± 1.43 9.66 ± 1.70	44.71 ± 8.23 45.52 ± 9.58	CKD-EPI
Liu 2019 [43]	Prospective cohort study	China	Allopurinol Febuxostat	96/112	53.40 ± 14.95 51.79 ± 13.54	36.5%/26.8%	6 months	3–5	8.93 ± 1.74 8.91 ± 1.52	28.06 ± 16.54 28.45 ± 16.55	CKD-EPI
Yang 2020 [44]	Retrospective cohort study	China	Allopurinol Febuxostat	83/233	65.73 ± 14.49 67.91 ± 13.38	26.5%/33.0%	6 months	3–5	9.37 ± 1.60 9.50 ± 1.53	40.75 ± 12.65 29.69 ± 13.84	NR
Lai 2022 [45]	Prospective cohort study	China	Febuxostat Placebo	32/34	88.66 ± 2.88 89.68 ± 3.35	43.7%/55.9%	3 months	3–4	8.31 ± 0.96 8.27 ± 1.04	42.91 ± 9.79 41.54 ± 6.91	CKD-EPI

*Median (IQR), RCT: random controlled trial, NR: not report, CKD-EPI: Chronic Kidney Disease Epidemiology Collaboration creatinine equation, MDRD: Modification of Diet in Renal Disease Study equation.

The results of the risk of bias assessment are presented in (Supplementary Figure S1). Results of RoB-2, 6 studies (50%) were evaluated as having some concerns, 4 studies (33.3%) showed a low risk of bias and 2 studies (open-label trials) had a high risk due to a lack of allocation concealment and blinding of participants and personnel. Additionally, the results of NOS, 3 studies (75%) were rated as high quality and 1 as moderate quality (Supplementary Table S3).

### 3.2. Primary outcomes

#### 3.2.1. The change of estimated glomerular filtration rate

A total of ten RCTs [21–24,34,37–41] and four cohort studies [42–45] involving 1,917 patients reported the effects of ULT on the changes in eGFR from baseline. For RCTs, the direct comparison showed that febuxostat (MD, 4.14 mL/min/1.73 m^2^; 95%CI, 0.65 to 7.63) and allopurinol (MD, 1.20 mL/min/1.73 m^2^; 95%CI, −1.22 to 3.62) had a higher eGFR level compared with placebo, but the latter was not significant (Supplementary Table S4). Two studies [23,40] compared febuxostat with allopurinol, the results showed that febuxostat was significantly associated with the higher eGFR level compared with allopurinol (MD, 6.58 mL/min/1.73 m^2^; 95%CI, 0.95 to 12.20) (Supplementary Table S4). However, the synthesized results of NMA demonstrated that there was no statistically significant association of febuxostat with allopurinol, although patients used febuxostat had higher eGFR than allopurinol (MD, 4.99 mL/min/1.73 m^2^; 95%CI, −0.65 to 10.78) (Figure 2B). Based on SUCRA values, febuxostat (97.7%) ranked first for improving kidney function (Supplementary Table S5). The node-splitting method revealed no inconsistency between direct and indirect evidence (Supplementary Table S6). The comparison-adjusted funnel plot indicated possible asymmetry (Supplementary Figure S2), and Egger’s test (*p* = 0.04) revealed a significant difference among the studies, suggesting a high risk of publication bias. After applying the trim and fill adjustment, no material change was observed in the result (Supplementary Figure S4). For cohort studies, three studies [42–44] involving 590 patients compared the effect of febuxostat and allopurinol on eGFR. Despite the 95%CI including the null value, the pooled results demonstrated that eGFR changes in febuxostat group were higher than those in allopurinol group (MD, 3.35 mL/min/1.73 m^2^; 95%CI, −1.55 to 8.25) (Supplementary Table S4). Another cohort study [45] compared febuxostat and placebo, the results showed that febuxostat treatment significantly increased the eGFR level from baseline to the end of follow-up (MD, 4.10 mL/min/1.73 m^2^; 95%CI, 1.10 to7.10).

#### 3.2.2. Major adverse cardiovascular events

Three RCTs [21,22,37] and two cohort studies [42,44] with 1,311 patients reported 108 MACE. The direct comparison results from RCTs indicated febuxostat did not significantly increase the risk of MACE compared with placebo (OR, 1.24; 95%CI, 0.18 to 8.40) (Supplementary Table S4). Only one RCT [22] showed that compared with placebo, a lower likelihood of developing MACE in allopurinol group, but there was no significance (OR, 0.69; 95%CI, 0.42 to 1.15). It should be pointed out that Jalal [35] reported one patient died of heart disease, which was considered to be related to the allopurinol treatment. Notably, both the NMA synthesized results of the RCTs and the direct comparison of cohort studies showed no significant difference in the risk of MACE between allopurinol and febuxostat (for RCTs, OR, 0.62; 95%CI, 0.06 to 4.99; for cohort studies, OR, 0.51; 95%CI, 0.05 to 5.22) (Figure 2B, Supplementary Table S4).

### 3.3. Secondary outcomes

#### 3.3.1. Change in serum uric acid from baseline

Ten RCTs [21–24,34,35,37,39–41] and four cohort [42–45] studies involving 1,915 patients reported the change in the SUA from baseline. For RCTs, the direct comparison and the NMA synthesized results both indicated that febuxostat was significantly associated with the lower SUA level than allopurinol (MD, −0.86 mg/dL; 95%CI, −1.17 to −0.55; and MD, −0.61 mg/dL; 95%CI, −1.15 to −0.05, respectively) (Figure 2C and Supplementary Table S4). Additionally, febuxostat (99.0%) ranked first for urate-lowering efficacy based on SUCRA values (Supplementary Table S5). The node-splitting method revealed no inconsistency between direct and indirect evidence (Supplementary Table S6). The comparison-adjusted funnel plot indicated possible asymmetry, whereas Egger’s test (*p* = 0.18) revealed no significant difference among the studies, suggesting a low risk of publication bias (Supplementary Figure S3). For cohort studies, three studies [42–44] reported direct compared febuxostat with allopurinol, the pooled result showed that febuxostat was significantly associated with the lower SUA level compared with allopurinol (MD, −1.79 mg/dL; 95%CI, −2.47 to −1.11) (Supplementary Table S4). Only one prospective cohort study [44] compared the febuxostat with placebo, the result indicated that febuxostat has the lower SUA level from baseline to the end of follow-up (MD, −3.29 mg/dL; 95%CI, −3.67 to −2.91).

#### 3.3.2. Change in urinary albumin/creatinine ratio

Only four RCTs [22,38,40,41] reported the change in ACR from baseline. Two RCTs [22,38] both indicated that there were no significant differences in the ACR between allopurinol and placebo (geometric mean difference, −9%; 95%CI, −24 to 10). Additionally, Nata et al. [41] reported that there were no significant differences on change of ACR from baseline between the febuxostat and placebo groups (median change (IQR): 0 (−109, 140.1) vs. 0.1 (−117, 45.6), *p* = 0.725). Matta et al. [40] reported the patients used febuxostat showed lower ACR values compared with allopurinol at the end of 4th month from baseline (−178.07 µg/mg creatinine vs. −140.04 µg/mg creatinine), but no significant differences between drugs.

#### 3.3.3. Adverse events

Eight RCTs [21,22,24,34,35,37,39,41] and three cohort studies [43–45] with 1,874 patients reported 525 AEs. The direct comparison of eight RCTs showed no significant difference in the risk of AEs across febuxostat and allopurinol treatment compared with placebo (OR, 2.01; 95%CI, 0.82 to 4.91; OR, 0.95; 95%CI, 0.52 to 1.72, respectively) (Supplementary Table S4). Moreover, the synthesized NMA results demonstrated there was no significant differences in the risk of AEs between allopurinol and febuxostat (OR, 0.31; 95%CI, 0.03 to 1.89) (Figure 2C). Two cohort studies [43,44] compared febuxostat with allopurinol, the pooled results showed that allopurinol was associated with a higher risk of AEs (OR, 1.45; 95%CI, 0.64 to 3.27) (Supplementary Table S4).

### 3.4. Heterogeneity, subgroup, sensitivity, and transitivity analysis

For synthesized results in outcomes with substantial heterogeneity, the subgroup analyses were conducted by different follow-up time and CKD stage. For the outcome of the change in eGFR from baseline, the pooled results indicated that febuxostat was associated with higher eGFR changes compared with allopurinol at 3 months (MD, 1.98 mL/min/1.73 m^2^; 95%CI −2.42 to 6.78), 6 months (MD, 7.75 mL/min/1.73 m^2^; 95%CI −1.37 to 17.39), and 18 months (MD, 1.99 mL/min/1.73 m^2^; 95%CI −0.77 to 4.74), but no statistically significant differences between drugs. Notably, the direct comparison results demonstrated that febuxostat was significantly associated with higher eGFR than allopurinol only at 4 (MD, 3.94 mL/min/1.73 m^2^; 95%CI 3.10 to 4.78) and 6 months (MD, 9.70 mL/min/1.73 m^2^; 95%CI 6.30 to 13.10) (Supplementary Table S7). Similarly, the pooled direct comparison results of two cohort studies showed that the eGFR change in febuxostat group was significantly higher than that in allopurinol group at 6 months follow-up (MD, 5.57 mL/min/1.73 m^2^; 95%CI 3.22 to 8.32). In terms of the CKD stage 3, the mixed RCT results only showed that febuxostat had higher eGFR level than allopurinol at 3 months (MD, 2.86 mL/min/1.73 m^2^; 95%CI −3.60 to 9.80) and 6 months (MD, 7.08 mL/min/1.73 m^2^; 95%CI −6.45 to 20.88), but no statistical significance was found. Two cohort studies [42,43] involving 185 patients compared the effect of febuxostat and allopurinol on eGFR, and the pooled results showed that in patients with CKD stage 3, the eGFR change from baseline in febuxostat group was significantly higher than in allopurinol group at 6 months of treatment (MD, 7.83 mL/min/1.73 m^2^; 95%CI 3.44 to 12.22). Only one cohort study [43] compared the effects of allopurinol and febuxostat on eGFR in CKD stage 4–5 patients. The results showed that both febuxostat and allopurinol reduced eGFR, especially in CKD 4 patients, allopurinol reduced eGFR more significantly than febuxostat (*p*﹤0.05).

Furthermore, the NMA synthesized results indicated that there were no significant differences between febuxostat and allopurinol in reducing the SUA level at 1 (MD, −0.27 mg/dL; 95%CI, −1.61 to 1.07), 2 (MD, 0.02 mg/dL; 95%CI, −0.82 to 0.83), 3 (MD, −0.16 mg/dL; 95%CI, −0.95 to 0.67), and 6 (MD, −0.59 mg/dL; 95%CI, −1.99 to 0.58) months of treatment, respectively, however, febuxostat showed more effective point estimated. The same findings were similarly fitted in the direct comparisons, except for the follow-up time was 6 months, the result showed that febuxostat significant decreased SUA levels (MD, −0.94 mg/dL; 95%CI, −1.40 to −0.48) (Supplementary Table S7). Additionally, the pooled direct comparisons of three cohort studies indicated that febuxostat was significantly associated with the lower SUA level compared with allopurinol at 1 month (MD, −1.06 mg/dL; 95%CI, −2.03 to −0.09), 3 months (MD, −1.10 mg/dL; 95%CI, −1.74 to −0.47), and 6 months (MD, −1.79 mg/dL; 95%CI, −2.47 to −1.11) of treatment. In terms of the CKD stage 3, the mixed results showed that febuxostat had lower SUA level than allopurinol at 3 months (MD, −0.94 mg/dL; 95%CI, −3.68 to 1.89) and 6 months (MD, −1.57 mg/dL; 95%CI, −5.15 to 1.99), but no statistical significance was found. Only one RCT [36] compared allopurinol to placebo at CKD stage 4, the results showed a significant decrease in SUA level during the 12-month from baseline administration of allopurinol (−1.49 ± 0.87) than placebo (−0.23 ± 0.89) (*p* = 0.02). Although we found the heterogeneity decreased owing the different treatment times, the heterogeneity still exists; however, there was insufficient information to perform analyses of the different dosage of febuxostat and allopurinol.

In addition, to assess the robustness of the pooled results, we conducted sensitivity analyses by excluding trials evaluated as having a high risk of bias overall. The NMA synthesized results showed that there was no significant association of febuxostat with the lower SUA level compared with allopurinol (MD, −0.49 mg/dL; 95%CI, −1.27 to 0.36). For other outcomes, no material change was observed in results or conclusions. According to the meta-regression results, similarities in clinical characteristics (age, female ratio, eGFR formulation, and racial) were observed across all the included studies, which implies that the transitivity of the trials was acceptable (Supplementary Table S8).

### 3.5. Quality of evidence

The confidence in the estimates for outcomes by RCTs was rated as moderate to very low (Supplementary Table S9). The major reason for downgrading the certainty of evidence was the risk bias, imprecision of the results with wide CIs, and heterogeneity.

## 4. Discussion

To the best of our knowledge, this is the first comprehensive network meta-analysis to summarize the comparative efficacy and safety of febuxostat and allopurinol in asymptomatic hyperuricemia patients with CKD stage 3–5. Our study demonstrated that compared with placebo, febuxostat and allopurinol both improve kidney function, particularly in significantly reducing SUA levels. Notably, moderate-to-low certainty evidence indicated that febuxostat was more effective than allopurinol in elevating eGFR levels and decreasing SUA levels, especially at 6 months of treatment. Although there were no significant differences in improving renal function between the two drugs in CKD stage 3 patients at 3 months and 6 months, febuxostat showed more effective point estimates. A previous meta-analysis also showed that compared to placebo, the febuxostat group showed a higher eGFR at 6 months [46]. Additionally, the two drugs do not significantly increase the risk of safety outcomes (MACE and other AEs). Asymptomatic patients are often neglected for treatment, and our results provide comprehensive evidence for treating those patients to attain better renal function.

Allopurinol and febuxostat both were xanthine oxidase inhibition; however, they differ in their mechanisms of action. Allopurinol mainly inhibits reduced xanthine oxidase; however, febuxostat inhibits both reduced xanthine oxidase and oxidized xanthine oxidase, making febuxostat was more effective than allopurinol in lowering the uric acid levels, especially in patients with hyperuricemia [47,48]. Previous meta-analysis had demonstrated better efficacy of febuxostat compared with allopurinol in treating hyperuricemia among kidney transplant patients [49]. Notably, an exploratory, retrospective, observational study has demonstrated that febuxostat effectively reduced serum uric acid concentrations and suppressed progressive decline in renal function in patients with CKD stage 3–5 who had failed treatment with allopurinol [50]. On the other hand, studies of animal models have shown that febuxostat suppressed renal ischemia-reperfusion injury and renal interstitial inflammation and fibrosis *via* reduced oxidative stress [51]. Thus, the renoprotective effect of febuxostat may not only be due to its potent uric acid-reducing effect to delay renal function progression, but also to the inhibition of oxidative stress through the inhibition of xanthine oxidase in pathological conditions of reduced renal function. Additionally, febuxostat is mainly excreted through renal and fecal routes, and requires no dose limitation in CKD stages 1–3. Our subgroup analysis of the CKD stage 3 revealed that febuxostat was associated with lower SUA level than allopurinol, whereas only one prospective cohort study [43] showed febuxostat was significantly associated with higher eGFR level than allopurinol in asymptomatic hyperuricemia patient with CKD stage 4 (−1.15 ± 6.02 mL/min/1.73 m^2^ vs. −5.50 ± 5.55 mL/min/1.73 m^2^, *p* < 0.05) and no significant difference was found in patients with CKD stage 5. Previous meta-analysis also showed that febuxostat has urate-lowering efficacy and no renal safety signal in patients with hyperuricemia and CKD stage 4–5 [52]; however, the number of studies and patients was relatively small and the included studies were observational study with a low level of evidence, these conclusions should be interpreted cautiously, considering the sparse data available. Similarly, two RCTs reported the safety and efficacy of febuxostat in patients with gout and moderate to severe renal impairment, the results showed that febuxostat led to significantly greater proportions of patients achieving a SUA level of <6.0 mg/dl compared with placebo [53,54]. On the other hand, the 2019 Chinese guidelines recommend a starting dose of 20 mg/d of febuxostat for all CKD patients, and prioritize febuxostat for uric acid-lowering intervention in patients with CKD stage 4–5 [18]. However, future well-designed trials with larger sample sizes to direct compare about allopurinol and febuxostat in patients with hyperuricemia with CKD stage 4–5 are needed to validate these results.

The CARES trial highlighted cardiovascular safety concerns regarding febuxostat, prompting a public safety alert from the FDA [55]. By contrast, a prospective, randomized, open-label, blinded-endpoint trial required by the European Medicines Agency (EMA), comparing febuxostat (80–120 mg/day) with allopurinol, did not support the finding of increased cardiovascular risk associated with febuxostat [56]. Our synthesized results showed no significant differences between allopurinol and febuxostat in increasing the risk of AEs and MACE, which were consistent with the recent published meta-analysis [57,58]. Furthermore, a multicenter, prospective, randomized open-label study (FREED) showed that febuxostat significantly reduced the risk of adverse cardiovascular and cerebrovascular events in patients with asymptomatic hyperuricemia compared with non-febuxostat group (HR 0.75, 95%CI 059 to 0.95, *p* = 0.017), and delayed the progression of renal dysfunction [59]. Although these studies indicated that neither allopurinol nor febuxostat increases or decreases the risk of MACE, febuxostat treatment of chronic hyperuricemia in patients with preexisting major cardiovascular diseases should be exercised cautiously, with particular caution in patients with evidence of high urate crystal and tophi burden or those initiating urate lowering therapy.

This study had some limitations to consider. First, the number of the included studies was too few to perform analyses of the different dosage of febuxostat and allopurinol. Second, more than 67% of trials included fewer than 60 patients per group, which may bring impression in synthesized results due to small sample size and downgrade in the GRADE assessment. Third, considering insufficient data in some outcome measures, we were unable to identify high homogenous through subgroup analyses, which need a larger number of studies with well-powered sample sizes to provide more definitive conclusions.

In conclusion, moderate-to-low certainty evidence indicated that febuxostat might benefit the enhancement of kidney function in asymptomatic hyperuricemia patients with CKD stage 3–5 compared to allopurinol or placebo, with equivalent safety profiles. Due to the limitations of the available data, further well-designed studies with larger sample sizes and detailed dosage information are needed to strengthen these findings.

## Supplementary Material

Revised Supplementary materials.docx

## Data Availability

Relevant data are within the article and supplementary materials.
